# Evaluating Spanish Translations of Emergency Department Discharge Instructions by a Large Language Model: Tool Validation and Reliability Study

**DOI:** 10.2196/79676

**Published:** 2026-01-12

**Authors:** Jossie A Carreras Tartak, Ryan CL Brewster, Daniela Arango Isaza, Antonio Berumen Martinez, Ana Grafals, Phanidhar Adusumilli, Ted Fitzgerald, Roger Orcutt, Larry A Nathanson, Adrian D Haimovich

**Affiliations:** 1 Department of Emergency Medicine Beth Israel Deaconess Medical Center Boston, MA United States; 2 Department of Pediatrics Beth Israel Deaconess Medical Center Boston, MA United States; 3 Department of Medicine Beth Israel Deaconess Medical Center Boston, MA United States; 4 Department of Technology and Innovation Beth Israel Lahey Health Boston, MA United States

**Keywords:** artificial intelligence, machine learning, machine translation, language, disparities

## Abstract

When given a sample of 100 emergency department discharge instructions, Claude Sonnet, a large language model, produced accurate Spanish translations as evaluated by Spanish-speaking physicians and medical interpreters.

## Introduction

Language-concordant emergency department (ED) discharge instructions are an essential component of equitable care for patients who prefer a language other than English [1-4]. ED discharge instructions are often complex, combining standardized templates with personalized clinician-written text. In most cases, patients who prefer a language other than English still receive instructions in English [5]. When translation is attempted, clinicians will often informally rely on tools such as Google Translate that are not auditable, are generally not institutionally approved for clinical use, and have known performance limitations for long or technically detailed documents [6-8].

Large language models (LLMs) offer a promising auditable and institutionally governable approach to addressing this equity gap [6,9]. Because reproducibility in patient-care processes requires controlled models rather than open chat interfaces such as ChatGPT (OpenAI), institutions will need to deploy translation LLMs directly [6]. To our knowledge, no formative pilot studies have evaluated LLMs for translating ED discharge instructions. We therefore conducted a feasibility study using real ED discharge instructions to assess LLM translation performance in preparation for clinical implementation.

## Methods

### Study Design

This was a single-center feasibility study at an urban academic medical center. We iteratively developed a translation prompt using Claude Sonnet (version 3.5; Anthropic) accessed via protected health information (PHI)–compliant Amazon Web Services (Amazon), testing each version on batches of 10 to 20 randomly sampled free-text discharge instructions provided to ED patients between July 1 and December 31, 2024. Claude Sonnet 3.5 was selected as it balances cost, performance, and speed and was available in our PHI-compliant environment.

Following prompt development, a team of independent evaluators (2 native Spanish-speaking physicians and 2 certified medical interpreters) reviewed a translated set of 100 randomly sampled free-text discharge instructions. We used a rubric adapted from prior studies consisting of a 5-point Likert scale across 5 domains (Multimedia Appendix 1) designed so that items rated a 3 or lower would be deemed substandard or unacceptable for use in a clinical setting [9]. Scores of 3 or lower in any domain required written explanation and were escalated for further review.

Our primary outcome was the proportion of discharge instructions that scored a 3 or lower in any one domain. The secondary outcome was the mean Likert score for each of the 5 domains stratified by reviewer type (interpreter vs physician). Descriptive analyses were performed in Python (version 3.11).

### Ethical Considerations

This study was approved by the Beth Israel Deaconess Institutional Review Board (2024P000315). All abstracted data were deidentified.

## Results

Of the 100 samples translated using the designed prompt (Multimedia Appendix 2), the mean Likert score ratings across samples by domain were as follows: 5.0 (95% CI 5.0-5.0) for completeness, 4.8 (95% CI 4.8-4.8) for fluency, 4.9 (95% CI 4.9-4.9) for meaning, 5.0 (95% CI 5.0-5.0) for severity, and 4.9 (95% CI 4.9-4.9) overall (Table 1; example translations are provided in Multimedia Appendices 3 and 4). One sample was given a score of 3 by a single reviewer in the domains of meaning and overall quality because the term “concussion” was translated as *conmoción cerebral* (full redacted translation in Multimedia Appendix 3). On adjudication, the translation was deemed clinically acceptable because the term *conmoción cerebral* is one of several translations of the term “concussion,” along with *concusión*.

**Table 1:** Interpreter and physician evaluator scores for Spanish translations (N=100).

Domain	Mean interpreter scores (95% CI)	Mean physician scores (95% CI)
Completeness	5.0 (5.0-5.0)	5.0 (5.0-5.0)
Fluency	4.8 (4.7-4.9)	4.8 (4.7-4.9)
Meaning	5.0 (5.0-5.0)	4.9 (4.9-4.9)
Severity	5.0 (5.0-5.0)	5.0 (5.0-5.0)
Overall	5.0 (5.0-5.0)	4.9 (4.9-4.9)

## Discussion

In this feasibility pilot study, we found that Claude Sonnet produced clinically acceptable Spanish translations of ED discharge instructions. The one case flagged for further review reflected regional differences in Spanish vocabulary, an observation suggesting that future LLM prompts may incorporate patient nationality or dialects to improve comprehensibility.

Our results are in alignment with prior work on standardized discharge instructions as well as free-text instructions from pediatric settings [9,10]. Free-text instructions have the potential for grammatical errors, dictation and typographical errors, missing information, formatting issues, and use of overly complicated medical terminology that might compromise translation quality. A recent study (n=20) in the pediatric setting showed comparable quality between interpreter translation and the GPT-4o model from OpenAI [10]. Our study did not directly compare the LLM outputs to interpreter outputs, but instead included interpreters as reviewers.

Our single-center results may not apply to institutions that have different discharge instruction processes or lack access to PHI-compliant LLMs. Moreover, our study was limited to Spanish. Further testing will be needed to establish the safety of LLM translation before live implementation.

## Data Availability

The datasets generated or analyzed during this study are available from the corresponding author on reasonable request.

## Appendix

Multimedia Appendix 1 Interpretation rating guide.

Multimedia Appendix 2 Large language model prompt.

Multimedia Appendix 3 Translation with low score.

Multimedia Appendix 4 Sample translation.

## Abbreviations

ED: emergency department
LLM: large language model
PHI: protected health information
