# Supplementary material for: Evaluating Spanish Translations of Emergency Department Discharge Instructions by a Large Language Model: Tool Validation and Reliability Study
Source: JMIR Form Res. 2026 Jan 12;10:e79676. doi: 10.2196/79676 (PMC12835839; doi:10.2196/79676)
Supplement: Multimedia Appendix 1 [file formative_v10i1e79676_app1.docx]

**Evaluator Instructions**

You have been asked to help evaluate discharge instructions translated by a large language model. Translations will be rated according to a 5-point Likert scale across the following domains (Figure 1):

1. Completeness: Does the translation preserve the original information?
2. Fluency: Is the translation grammatically accurate?
3. Meaning: Is the original intention and connotation maintained?
4. Severity: What is the risk of causing significant harm or delays in care?
5. Overall Quality: Taking the above factors into consideration, what is your overall rating the translation?

Differentiating *completeness* and *meaning*: Completeness refers to the extent to which the literal information and language of the original is preserved while meaning assesses whether translation maintains the intent of the original text.

Using the example of “take the medication prescribed twice a day”.

A translation that says “take the medication prescribed” has acceptable meaning, but lower completeness.

A translation that says “take the medication every two days” has acceptable completeness, but lower meaning.

**Evaluation Rubric**

|  | **Adequacy** | **Completeness** | **Meaning** | **Severity** | **Overall Quality** |
| --- | --- | --- | --- | --- | --- |
| 1 | 0% of information conveyed from the original | No fluency; no appreciable grammar, not understandable | Completely different from the original | Clearly dangerous to patient | Poor |
| 2 | 25% of information conveyed from the original | Marginal fluency; several grammatical errors | Slightly the same meaning as the original | Potential to be dangerous to patient | Fair |
| 3 | 50% of information conveyed from the original | Good fluency; several grammatical errors, understandable | Partially the same meaning as the original | Has effect on patient care but not dangerous | Good |
| 4 | 75% of information conveyed from the original | Excellent fluency; few grammatical errors | Almost the same meaning as the original | Unclear effect on patient care | Very Good |
| 5 | 100% of information conveyed from the original | Perfect fluency; like reading a newspaper | Same meaning as the original | No effect on patient care | Excellent |

**Note:** For any item scoring a 3 or less, please write a comment as to why you are assigning that score.

In addition, to determine to what degree any issues with translation might be attributable to the quality of the original English instructions, we are also asking you to evaluate the original instructions with a single question:

**How clear and easy to understand are the original (English) discharge instructions for a typical patient?**

1: Very unclear, difficult to understand

2: Somewhat unclear, needed to reread parts

3: Neutral, instructions were mostly clear

4: Mostly clear, only a few minor confusions

5: Extremely clear, easily understood with no confusion
