# Supplementary material for: Evaluating Spanish Translations of Emergency Department Discharge Instructions by a Large Language Model: Tool Validation and Reliability Study
Source: JMIR Form Res. 2026 Jan 12;10:e79676. doi: 10.2196/79676 (PMC12835839; doi:10.2196/79676)
Supplement: Multimedia Appendix 3 [file formative_v10i1e79676_app3.docx]

**Translation flagged by single evaluator**

**Original English text:**

“You were evaluated today after a head injury. The cause of your symptoms today is most consistent with a concussion. A concussion is a mild brain injury that can cause symptoms such as confusion, memory loss, nausea, and headache. Sometimes people pass out (lose consciousness) when they have a concussion, but not always. Concussions do not show up on any imaging tests such as a CT scan or MRI. They are diagnosed based on your symptoms and examination. Fortunately there was no evidence of a serious brain injury on your exam.

A concussion does not usually need treatment. Most concussions get better on their own, but it can take time. Some people's symptoms go away within minutes to hours. Other people have symptoms for weeks to months. When symptoms last a long time, doctors call it "post-concussion syndrome".

You finger numbness is likely related to a problem called Carpal Tunnel Syndrome we have referred you to the hand surgery team.

To help your recovery after a concussion, you can:

_Rest your body – Make sure to get plenty of sleep. Avoid heavy exercise or too much physical activity if it makes you feel worse. If light exercise does not make your symptoms worse, you can gradually increase your activity level. Please try to avoid any repeat head injuries. If you play contact sports, speak with your doctor before returning to your sport.

_Rest your brain – Avoid doing activities that need concentration or a lot of attention if they make you feel worse (eg, reading, screen time like TV/computer usage, focused work). You can start doing these things again as you get better.

_Do not drink alcohol while you are still having symptoms of concussion

_ For pain, you may take Ibuprofen (Motrin, Advil) 400mg every 6-8 hours as needed and/or Tylenol (Acetaminophen) 1000mg every 6-8 hours (Do not take more than 3000mg of Acetaminophen per day).

Please return or see your doctor immediately if:

_You vomit multiple times

_You have a severe headache, or a headache that gets worse

_You have a seizure or confusion

_You have trouble walking or talking

_Your vision changes

_You feel weak or numb in part of your body

_You lose control over your bladder or bowels

Or, please call your doctor or return if you have any concerns.”

**LLM Translation:**

“Se le evaluó hoy después de una lesión en la cabeza. La causa de sus síntomas es más consistente con una conmoción cerebral. Una conmoción cerebral es una lesión cerebral leve que puede causar síntomas como confusión, pérdida de memoria, náuseas y dolor de cabeza. A veces las personas se desmayan (pierden el conocimiento) cuando tienen una conmoción cerebral, pero no siempre. Las conmociones cerebrales no se muestran en ninguna prueba de imagen como la tomografía computarizada o la resonancia magnética. Se diagnostican según sus síntomas y el examen físico. Afortunadamente, no hubo evidencia de una lesión cerebral grave en su examen.

Una conmoción cerebral generalmente no necesita tratamiento. La mayoría mejora por sí sola, pero puede tomar tiempo. Los síntomas de algunas personas desaparecen en minutos u horas. Otras personas tienen síntomas durante semanas o meses. Cuando los síntomas duran mucho tiempo, los médicos lo llaman "síndrome post-conmoción cerebral".

El entumecimiento de sus dedos está probablemente relacionado con un problema llamado Síndrome del Túnel Carpiano y lo hemos referido al equipo de cirugía de mano.

Para ayudar a su recuperación después de una conmoción cerebral, puede:

_Descansar su cuerpo – Asegúrese de dormir lo suficiente. Evite el ejercicio intenso o demasiada actividad física si esto empeora su condición. Si el ejercicio ligero no empeora sus síntomas, puede aumentar gradualmente su nivel de actividad. Por favor, trate de evitar cualquier lesión adicional en la cabeza. Si practica deportes de contacto, hable con su médico antes de volver a su deporte.

_Descansar su cerebro – Evite realizar actividades que requieran concentración o mucha atención si estas empeoran su condición (por ejemplo, leer, tiempo frente a pantallas como TV/computadora, trabajo concentrado). Puede volver a realizar estas actividades a medida que mejore.

_No beba alcohol mientras tenga síntomas de conmoción cerebral

_Para el dolor, puede tomar ibuprofeno (Motrin, Advil) 400mg cada 6-8 horas según sea necesario y/o acetaminofén (Tylenol) 1000mg cada 6-8 horas (No tome más de 3000mg de acetaminofén por día).

Por favor, regrese o consulte a su médico inmediatamente si:

_Vomita varias veces

_Tiene dolor de cabeza severo o un dolor de cabeza que empeora

_Tiene una convulsión o confusión

_Tiene problemas para caminar o hablar

_Su visión cambia

_Siente debilidad o entumecimiento en alguna parte de su cuerpo

_Pierde el control de su vejiga o intestinos

O, por favor llame a su médico o regrese si tiene alguna inquietud.”

**Reviewer scores:**

| Reviewer | Completeness | Fluency | Meaning | Severity | Overall | Original |
| --- | --- | --- | --- | --- | --- | --- |
| Physician 1 | 5 | 5 | 5 | 5 | 5 | 4 |
| Physician 2 | 5 | 5 | 3 | 5 | 3 | 5 |
| Interpreter 1 | 5 | 5 | 5 | 5 | 5 | 5 |
| Interpreter 2 | 5 | 4 | 5 | 5 | 5 | 5 |

Comment by Physician 2: “Concussion is not the same as conmocion, meaning of the main diagnosis is not accurate in spanish translation here”
