# Supplementary material for: Evaluating Spanish Translations of Emergency Department Discharge Instructions by a Large Language Model: Tool Validation and Reliability Study
Source: JMIR Form Res. 2026 Jan 12;10:e79676. doi: 10.2196/79676 (PMC12835839; doi:10.2196/79676)
Supplement: Multimedia Appendix 4 [file formative_v10i1e79676_app4.docx]

**Original English text:**

“You are seen in the emergency department today for right-sided lower back pain that you have been experiencing for the last few months. As we discussed even though unfortunately your surgery was canceled we are unable to reschedule that here in the emergency department for you. You were given some medicine to help with your pain. As far as your high blood pressure goes we checked your labs which did not show anything concerning. We checked your urine and it showed nothing concerning

We have sent you home with a few days supply of pain medicines. Please take these as prescribed.

You may take Ibuprofen (Motrin, Advil) 400mg every 6-8 hours as needed. Do not take on an empty stomach. Stop taking this if you experience abdominal pain, or black or bloody stool.

You may take Tylenol (Acetaminophen) 1000mg every 6-8 hours. Do not take more than 3000mg per day.

At this time he feels that you are safe to discharge home with follow-up from your spine surgeon and primary care physician. If you continue to experience increased headaches, visual disturbances, chest pain, numbness in your groin, incontinence of urine or stool, or any other symptoms that concern you please return to the emergency department for further evaluation.”

**Spanish translation:**

“Usted fue atendido/a en el departamento de emergencias hoy por dolor en la parte baja derecha de la espalda que ha estado experimentando durante los últimos meses. Como discutimos, aunque lamentablemente su cirugía fue cancelada, no podemos reprogramarla aquí en el departamento de emergencias. Se le administró medicamento para ayudar con su dolor. En cuanto a su presión arterial alta, revisamos sus análisis de laboratorio que no mostraron nada preocupante. Examinamos su orina y no mostró nada preocupante.

Le hemos enviado a casa con un suministro de medicamentos para el dolor para algunos días. Por favor, tómelos según lo prescrito.

Puede tomar ibuprofeno (Motrin, Advil) 400mg cada 6-8 horas según sea necesario. No lo tome con el estómago vacío. Deje de tomarlo si experimenta dolor abdominal o heces negras o con sangre.

Puede tomar Tylenol (acetaminofén) 1000mg cada 6-8 horas. No tome más de 3000mg por día.

En este momento, se considera que es seguro darle de alta para que regrese a casa con seguimiento de su cirujano de columna y su médico de atención primaria. Si continúa experimentando dolores de cabeza intensos, alteraciones visuales, dolor en el pecho, entumecimiento en la ingle, incontinencia urinaria o fecal, o cualquier otro síntoma que le preocupe, regrese al departamento de emergencias para una evaluación adicional.”

**Reviewer scores:**

| Reviewer | Completeness | Fluency | Meaning | Severity | Overall | Original |
| --- | --- | --- | --- | --- | --- | --- |
| Physician 1 | 5 | 4 | 4 | 5 | 4 | 4 |
| Physician 2 | 5 | 5 | 5 | 5 | 5 | 5 |
| Interpreter 1 | 5 | 5 | 5 | 5 | 5 | 5 |
| Interpreter 2 | 5 | 5 | 5 | 5 | 5 | 5 |
